# Mesoscale Near-Surface Wind Speed Variability Mapping with Synthetic Aperture Radar

**DOI:** 10.3390/s8117012

**Published:** 2008-11-05

**Authors:** George Young, Todd Sikora, Nathaniel Winstead

**Affiliations:** 1 The Pennsylvania State University, Department of Meteorology / 503 Walker Building, University Park, PA 16802, U.S.A.; 2 Millersville University, Department of Earth Sciences / P.O. Box 1002, Millersville, PA 17551, U.S.A.; E-Mail: todd.sikora@millersville.edu; 3 Johns Hopkins University, Applied Physics Laboratory / 11100 Johns Hopkins Road, Laurel, MD 20723, U.S.A.; E-Mail: nathaniel.winstead@jhuapl.edu

**Keywords:** Synthetic Aperture Radar, Digital Filter, Pattern Recognition

## Abstract

Operationally-significant wind speed variability is often observed within synthetic aperture radar-derived wind speed (SDWS) images of the sea surface. This paper is meant as a first step towards automated distinguishing of meteorological phenomena responsible for such variability. In doing so, the research presented in this paper tests feature extraction and pixel aggregation techniques focused on mesoscale variability of SDWS. A sample of twenty eight SDWS images possessing varying degrees of near-surface wind speed variability were selected to serve as case studies. Gaussian high- and low-pass, local entropy, and local standard deviation filters performed well for the feature extraction portion of the research while principle component analysis of the filtered data performed well for the pixel aggregation. The findings suggest recommendations for future research.

## 1. Introduction

Images from satellite synthetic aperture radar (SAR) of the ocean surface have been shown to routinely reveal the sea-surface roughness signatures of marine meteorological phenomena [1, 2]. This is because the near-surface wind rapidly generates short surface waves (here, we are referring to the order of magnitude 1 to 10 centimeter wavelengths) that roughen the surface. The characteristic wavelength of the SARs referenced above is also order of magnitude 1 to 10 centimeters. For example, the SAR aboard the Canadian Space Agency's (CSA's) RADARSAT-1 satellite is a C-band system (wavelength of approximately 5 centimeters). At moderate incident angles, such as those employed in the studies referenced above (e.g., 20° to 49° for RADARSAT-1 ScanSAR Wide mode), the bulk of the SAR backscatter is due to surface roughness elements that scale with the radar wavelength projected onto the scattering surface (i.e., Bragg scattering, as opposed to specular and wedge scattering). Thus, for the ocean surface, those roughness elements include the centimeter-scale waves driven by the near-surface wind. It follows that the variability of normalized radar cross section (NRCS, average backscatter divided by area) on a SAR image of the ocean is a function of the coincident overlying meteorological phenomena. For a recent review of SAR principles, see [3].

**Figure 1.**
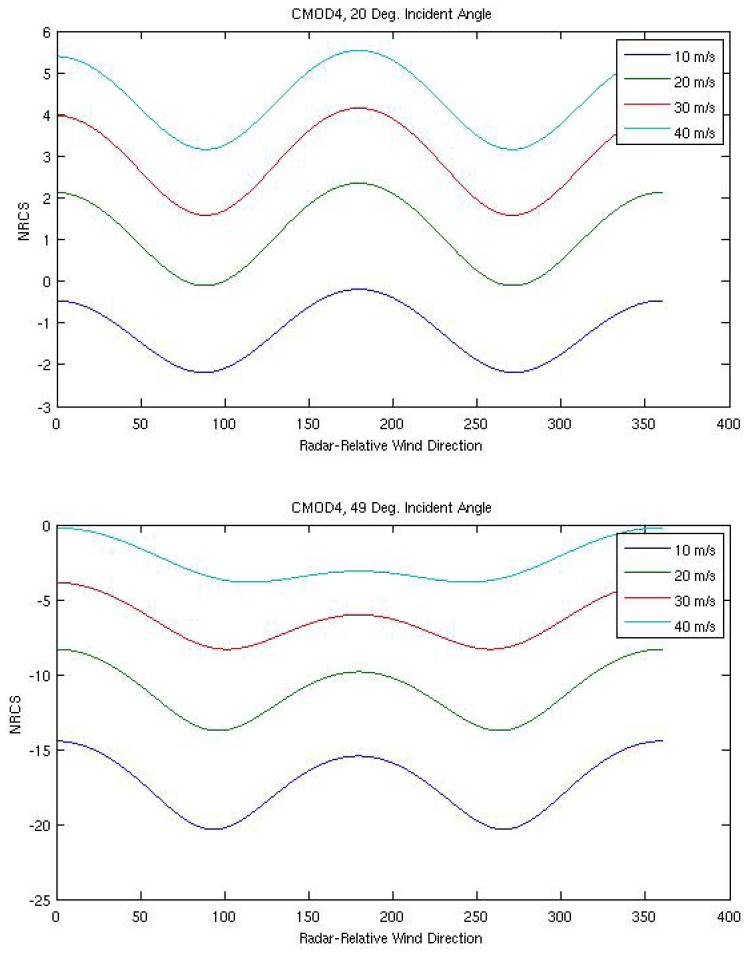

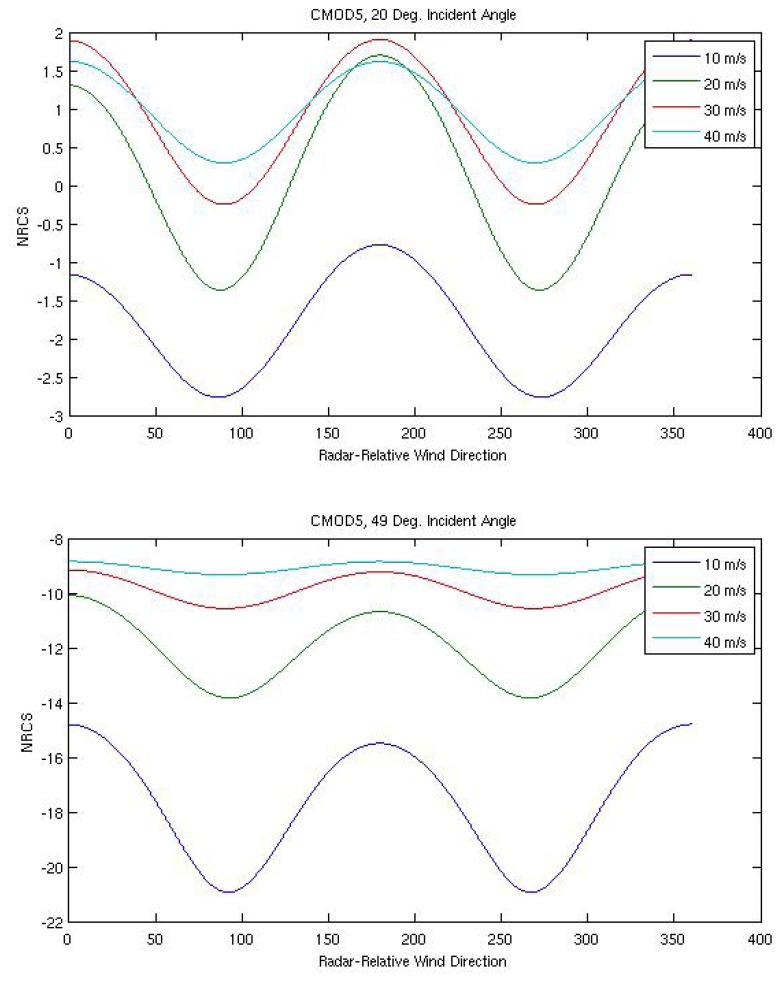
The relationship between NRCS, near-surface wind speed, and radar-relative near-surface wind direction for CMOD 4 and CMOD 5, continued on next page.

For the remainder of this discussion, we will focus on moderate incident angle wide-swath SAR images because they are the most commonly employed in corresponding meteorological studies. From the information provided above, one may conclude that as near-surface wind speed increases, so does SAR NRCS. However, there is a growing body of evidence that there is near-surface wind speed limit to this function, at which point the SAR NRCS becomes saturated [4]. This saturation is a function of incidence angle and polarization with HH-pol saturation occurring at a larger wind speed than VV-pol. The SAR NRCS is also a function of near-surface wind direction. Because the centimeter-scale surface waves driven by the near-surface wind generally travel with their crests oriented perpendicular to the near-surface wind direction, there are local maxima in NRCS when the radar look direction is opposite and along the near-surface wind direction. Local minima in NRCS occur when the radar look direction is perpendicular to the near-surface wind direction. The relative magnitudes of the maxima are incident angle and near-surface wind speed dependent. As incident angle increases (say, from 20° to 49°) and near-surface wind speed decreases (say, from 40 ms^-1^ to 10 ms^-1^), the NRCS becomes largest for a radar look direction opposite to the near-surface wind direction and there is a larger difference in NRCS between the maxima and minima. Figure 1 demonstrates these relationships from empirically-derived C-band geophysical MODel (CMOD) functions (GMF) 4 [5] and 5 [4]. Both GMFs are tuned to wind speed at 10 m above sea level. Within Figure 1, they have been modified from vertical-vertical (VV) polarization to horizontal-horizontal (HH) polarization (i.e., to that of RADARSAT-1). These GMFs will be discussed in more detail below.

Radar incident angle, wavelength, and polarization also affect the relationship between the near-surface wind vector and NRCS while sky condition (cloudy/clear, day/night) does not. As incident angle increases, NRCS decreases (see Figure 1). Of the typical wavelengths used for SAR, C-band provides the larger NRCS. VV polarization provides larger NRCS than HH polarization. And, of course, phenomena other than the near-surface wind vector can influence the spectrum of Bragg scatterers and therefore the NRCS (e.g., surfactant slicks, current shear, precipitation, and swell). See [6] for a thorough review of SAR imaging of the ocean surface.

The horizontal resolution of satellite SARs are quite high and their swath widths are large enough to capture the complete sea-surface roughness signatures of many meteorological phenomena. For example, the horizontal resolution for ScanSAR Wide images is 100 m with a swath width of 500 km. Other satellite SARs offer resolution 2 orders of magnitude greater than this (e.g., 1 m from that of the German Aerospace Agency's TerraSAR-X in SpotLight Mode and 3 m from that of the CSA's RADARSAT-2 in Ultra-Fine Mode), but at reduced geographic coverage (narrower swath widths).

The high-resolution, wide swath, all-weather operation, near-surface wind-sensitive nature of SAR has fueled its emergence as a meteorological tool. Early studies, such as those referenced in [1-2], employed scaled NRCS SAR images to support feature detection and analysis. For example, [7, 8-10] examined convection cells of various scales. [11, 12] studied atmospheric gravity waves. [13-15] focused on atmospheric roll vortices. [16-18] investigated polar mesoscale cyclones while [19, 20] did so for tropical cyclones. [21, 22] researched the SAR signatures of atmospheric fronts and their prefrontal jets. We stress that the references above do not represent an exhaustive list of research into atmospheric features examined with SAR. Moreover, a similar compendium of research exists for oceanic feature detection [e.g., 23, 6].

Because of the relationship between SAR NRCS and the near-surface wind vector, several research groups have endeavored to employ satellite SAR as a high-resolution (order of magnitude 0.1 to 1 km) scatterometer using an appropriate GMF (see the reviews by [24, 25]). Referring to the preceding discussion and Figure 1, note that given a SAR system of a particular wavelength and polarization, sensing an area of ocean at a given incident angle, over which there is a constant moderate near-surface wind speed, one must still ascertain, a priori, the corresponding near-surface wind direction before near-surface wind speed can be calculated from the NRCS. This represents a liability compared to traditional scatterometry. Traditional scatterometers overcome this problem by sensing a given area of ocean surface from more than one look direction near instantaneously through either rotating conical scanning beams (e.g. the SeaWinds scatterometer aboard the National Aeronautics and Space Administration QuikSCAT satellite) or fixed beams (e.g. the Advanced Scatterometer aboard the European Space Agency (ESA) MetOP satellite). Near-surface wind direction ambiguity may be further reduced via numerical weather prediction (NWP) models. The liability of traditional satellite scatterometry over SAR scatterometry is its relatively large grid spacing (order of magnitude 10 km).

The near-surface wind direction information used for SAR-derived wind speed (SDWS) have included NWP models [e.g., 26], scatterometers [e.g., 27], and linear geophysical features found within the SAR images [e.g., 28]. Comparisons between SDWS and that from buoys, NWP models, and scatterometers are quite good (root mean square errors of a couple ms^-1^ typically reported as in the papers cited above). It is also heartening to note that even though the routinely employed GMFs were validated over the open ocean, they have been found to be robust in the vicinity of mountainous coastlines [e.g., 29].

**Figure 2.**
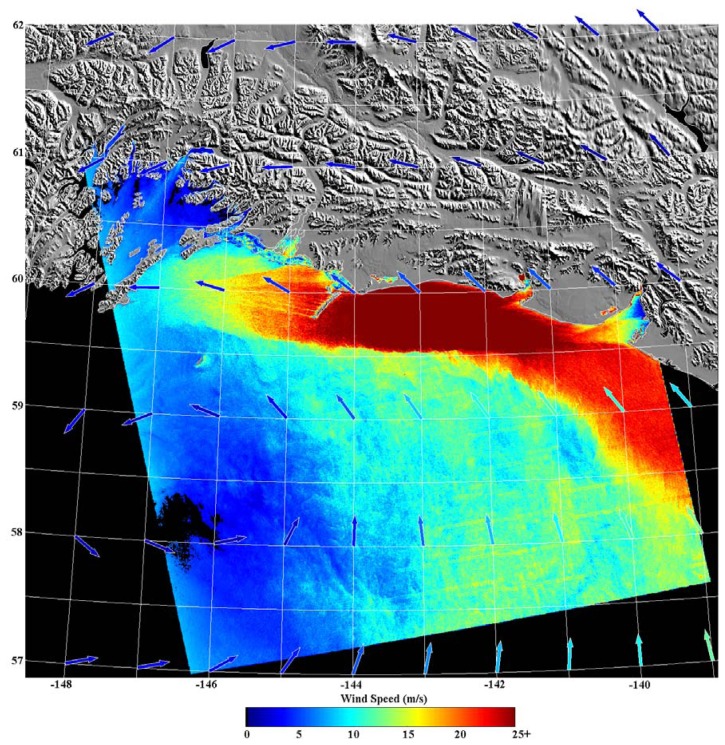
SDWS image from 0310 UTC on 18 February 2000 [2].

It follows that the meteorological phenomena sampled in NRCS SAR images are also present in corresponding SDWS images. [30] provides a wealth of such images and relevant citations. As an example of the detail available within SDWS images, consider Figure 2, which shows a SDWS image from 0310 UTC on 18 February 2000, originally described in [31]. The corresponding SAR image is from RADARSAT-1 ScanSAR Wide mode. The pixel size of the SDWS image is approximately 600 m and it was generated using CMOD 4 with near-surface wind directions from the United States Navy's NOGAPS NWP model. The NOGAPS wind vectors are the colored arrows within the image. The SDWS image is cropped at 25 ms-1 as that is near the accepted upper limit of applicability for CMOD 4. The 50 km band of large wind speed adjacent to the coast is a due to a mesoscale barrier jet forced by terrain blocking associated with a land-falling occluded front [32]. Other SDWS features of note include the sharp demarcation of the mesoscale barrier jet on its seaward side, gap flows [33] emanating from Icy Bay (60.00°N, 141.50°W) and Yakutat Bay (59.75°N, 140.00°W), and the variability in the wake [33] of Kayak Island (59.75°N, 144.4°W).

The near-surface wind speed variability revealed within Figure 2 (a change from breeze to gale/storm force over a distance of 1 to 10 km) is not atypical of SDWS images. It is clear that knowledge of the near-surface wind speed variability present within the imaged area of Figure 2 would be of great value to civilian and military maritime operations also within that area. Without the SDWS image, it is doubtful whether the variability referred to above would have been detected. Operational numerical weather prediction models can fail to capture the flow in the vicinity of complex terrain, as seen for example in the difference in wind direction between NOGAPS and the SAR-detected island wakes in Figure 2. Recall that the resolution of scatterometer data is coarser than that of SDWS images (order of magnitude of 10 km) and thus cannot sample the near-surface wind speed adjacent to the coast or its sub-resolution variability. Finally, there were only two operational buoys within the image area, both located within the northeastern portion of the image.

The present research describes digital filtering and analysis techniques for SDWS images aimed at eventual automated distinguishing of the meteorological sources of mesoscale (defined by [34] as wavelengths of 2 km to 2000 km) near-surface wind speed variability. The description of the SDWS data is located in Section 2. Section 3 contains the image filtering and analysis procedures employed. Key results from a set of 28 case studies are found in Section 4. Section 5 presents conclusions and recommendations for future work.

## 2. Data

The SDWS data presented herein are based on a selection of RADARSAT-1 SAR images containing various commonly observed meteorological phenomena. A total of 28 RADARSAT-1 ScanSAR Wide B images (pixel spacing as small as 50 m and a swath width of 450 km, as processed by the Alaska Satellite Facility (ASF)) were selected as cases for this study. All cases are images from Alaskan coastal waters including the Gulf of Alaska, the Bering Sea, and the waters surrounding the Aleutian Islands. These regions are areas where strong forcings commonly occur and interact to generate significant spatial near-surface wind speed variability. Recall that in the present research, we focus on mesoscale near-surface wind speed variability. It is often the case that multiple overlapping mesoscale meteorological phenomena occur within one 450 km swath. Here, the choice of images was carefully designed to provide a robust test for the filtering techniques described within Section 3. The SAR images were downloaded from the National Oceanic and Atmospheric Administration's (NOAA's) Satellite and Information Service Comprehensive Large Array-data Stewardship System (CLASS) and are available at http://www.nsof.class.noaa.gov/saa/products/welcome.

### 2.1 Image Processing

The system used to convert all of the SAR images to SDWS for the present research is the Johns Hopkins University Applied Physics Laboratory (APL), NOAA, SAR Wind Retrieval System (ANSWRS) software package, described in [35]. This software package was developed to provide a means of quickly converting SAR images to SDWS images by assuming *a priori* knowledge of the near-surface wind direction. The operational version of ANSWRS at APL, NOAA, and the ASF employs NOGAPS near-surface wind directions. ANSWRS is capable of processing satellite SAR images from RADARSAT-1 and RADARSAT-2, ESA's ERS-2 and ENVISAT, the Japanese Aerospace Exploration Agency's Advanced Land Observing Satellite, and TerraSAR-X. The GMFs CMOD 4 and CMOD 5 as well as L-band and X-band GMFs are available with the ANSWRS package. In addition to NOGAPS wind directions, those from other NWP models, and generic user-specified and SAR-based techniques, are also available. The outputs from ANSWRS include SDWS images and netCDF files containing the SDWS data.

The parent ScanSAR Wide B images employed herein were processed by ANSWRS. A brief summary of the configuration follows. First, 3 pixel by 3 pixel averaging was performed on the calibrated raw NRCS images in order to optimize the reduction of speckle while preserving maximum meteorological variability. The images were then converted to wind speed using NOGAPS near-surface wind directions and CMOD 4. Each SDWS image was then resampled onto a rectangular latitude – longitude grid. The final image size for all images processed here is 1200 × 1200 pixels and the final pixel size is approximately 450 m. Table 1 provides a summary of each case study image of the present research.

## 3. Procedures

The SDWS image analysis procedures described in this section are designed to explore the potential for automated discrimination between the various phenomena responsible for mesoscale variability in near-surface wind speed over the ocean. Thus, at most stages in the process, several different methods are tested in parallel. Output from the most successful methods at each stage is used as the input for the next stage. As in most image recognition applications, the process is broken into four stages [36]. First the image data are cleaned to remove artifacts of no interest to the current analysis, in this case NRCS from land. Second, various two-dimensional filters are applied to each image as a means of feature extraction. Third, various aggregation algorithms are applied to the filtered images to diagnose which pixels belong to the same phenomenon. The results of the second two stages are assessed manually.

Finally, various artificial intelligence algorithms are described which could be applied in future studies to automatically determine which mesoscale atmospheric phenomenon is associated with each group of pixels in the SDWS image. Thus, only the first three stages are tested here due to the limited number of cases. All three stages were performed by a single Matlab program, written to exploit the modularity of the Matlab image processing, statistics, and mapping toolboxes (http://www.mathworks.com/).

### 3.1 Image Cleaning

The primary contaminant of SDWS images is NRCS from land. While it is possible to distinguish land from sea by statistical analysis of the SAR images, it is easier to use one of the readily available geographic datasets to mask out the land areas within the image. This masking is done in two stages, first to set the wind speed in any potentially land-contaminated pixels to a value of least regret, zero, and then to mask out the broader area which those zero values could impact through the two-dimensional feature extraction filters. For filters with a strictly finite radius of influence, the first stage is not necessary. It is included here to allow for filters with weak tails extending beyond their nominal radius of influence, although none such are used in this research. The first land masking stage takes place before feature extraction while the second land masking stage takes place after feature extraction.

For this research, the United States Geologic Survey (USGS) 30 minute gtopo30 digital elevation model (DEM) data were used. These data were first downloaded from the USGS web site (http://edc.usgs.gov/products/elevation/gtopo30/gtopo30.html) and then read into the Matlab analysis program and interpolated onto the same geospatial grid as the SDWS images using the mapping toolkit's ltln2val function. Because pixels that contain even some land are contaminated for the purposes of this analysis, the land mask is extended two pixels beyond those initially marked as land. This extension is done using the mapping toolkit's bwdist distance transform function with a chessboard distance metric,

(1) $$\text{Dist}=\max(\left|x_{1}-x_{2}\right|,\left|y_{1}-y_{2}\right|),$$

where x_1_ and y_1_ are the coordinates of one pixel and x_2_ and y_2_ those of the other.

The second stage of land masking is identical to the first except that it is applied to the filtered SDWS images and uses a buffer that is two pixels greater than the filters' radius of influence, the extra pixels again being included to allow for the spread in land contamination to pixels adjacent to those marked as land in the USGS dataset.

### 3.2 Feature Extraction

Image filtering for feature extraction is intended not to isolate a single characteristic that unambiguously identifies a particular atmospheric phenomenon, but rather to provide a set of metrics that will together provide enough information to distinguish between all of the phenomena on an image. The success of this process depends both on the degree to which the various phenomena exhibit different spatial patterns of near-surface wind speed variability and the ability of the filters to capture this set of distinctions. Because each of the atmospheric phenomena observed in the Gulf of Alaska SDWS images can be distinguished manually by a skilled meteorological analyst, the first criterion is met. Thus, the key issue is selection of an appropriate set of filters.

The filters tested in the present research are all implemented in the Matlab image processing toolbox. Each is a two-dimensional finite impulse response (FIR) filter. They include popular examples of each of the most common filter categories used in digital image analysis. The Matlab functions implementing each function are listed in Table 2.

For high and low-pass filtering, a Gaussian filter is used with a standard deviation of 10 pixels (3 km) and a radius of influence of 21 pixels (9.45 km). The low-pass Gaussian filter maps the mean near-surface wind speed while the high-pass Gaussian filter maps its mesoscale variability which is the target of this research. No one choice of filter radius is optimal for all mesoscale meteorological phenomena. Indeed, the choice used here appears to be too small for several of the phenomena found in the Gulf of Alaska (see the Results section).

A high-pass local entropy filter [37] is applied to quantify near-surface wind speed variability over a broader range of scales. The entropy value reported at each pixel is calculated over a rectangular neighborhood of 41 by 41 pixels, 18.45 by 18.45 km. Thus, it has the same influence radius as the Gaussian filter. A high-pass local standard deviation filter over the same neighborhood is applied to obtain a different measure of this same information. A local median filter provides a more scale-restricted measure of near-surface wind speed variability, focused on scales smaller than the radius of influence. It is applied here as a high-pass filter over the same neighborhood used with the local entropy filter.

Because none of the filters above distinguishes between edges and other morphologies of near-surface wind speed variation, an edge detection filter is also applied. The edge detection filter is applied with the Sobel [38] approximation to the near-surface wind speed gradient. The available alternative approximations (Pewitt, Roberts, Zero-Cross, Log, and Canny) were contemplated, but Sobel was found to yield the most nearly continuous string of flagged edge pixels for atmospheric wind speed boundaries.

The high-pass and edge detection filters provide a certain degree of redundancy, but also measure different aspects of the mesoscale near-surface wind speed variability as described above. The correlation matrix for the filters is shown in Table 3. The correlations are computed separately for each of the 28 test images, with the mean and standard deviations of these values being presented in the table. The correlation matrix indicates that none of these measures is completely redundant. The outputs of the entropy and median filters are highly correlated while those of the Gaussian high-pass filter and the Sobel edge detection filters are virtually independent of the other filter outputs. The correlations between the local standard deviation filter output and those of the local entropy and median filters are all modest but non-negligible.

The information extracted from the SDWS images by these filters could probably be increased by more extensive tuning of the filter radii. In particular, the use of band-pass filters to eliminate both the synoptic scale flow and the small scale noise would probably improve the feature extraction for mesoscale near-surface wind speed variability.

### 3.3 Pixel Aggregation

Two different approaches to pixel aggregation are tested: Principle Component Analysis (PCA, [39]) and Cluster Analysis [40]. Each is applied to the results of the feature extraction filters described above. PCA is tested because it is a traditional approach for reducing the degrees of freedom in a set of correlated variables. For unrotated or orthogonally rotated PCA, it also eliminates colinearity between the variables. PCA has two disadvantages for the current application. First, as with the clustering method described next, the rotated versions require that one specify the number of patterns to be searched for, in this case the number of components to be retained and rotated. Second, the rotation algorithms, whether orthogonal or oblique, are designed to maximize simple structure in the components, i.e. minimize the number of filter outputs that contribute significantly to each component. In this application it would be preferable to optimize instead for the spatial coherence of the resulting component scores, i.e. the areas of the image designated as belonging to a distinct mesoscale atmospheric phenomenon.

The output of the five filters described in Table 3 is used as input to the PCA. Experimentation suggests that there are usually two to four phenomena present in an image with operationally significant mesoscale near-surface wind speed variability. Therefore, four of the five resulting components are rotated. The Matlab functions used in this analysis, as well as the cluster analysis described below, are shown in Table 4. Varimax (orthogonal) and Promax (oblique) rotations are applied (Richman 1986). Examination of the principle component (PC) score maps (see the Results section) reveals that while each component quantifies a different aspect the mesoscale variability of the near-surface wind speed, multiple phenomena contribute to each component, even when rotated.

Clustering, in this case via the k-means algorithm, provides a potential solution to this aggregation task as it optimizes pixel groupings so that the pixels within each group closely resemble each other across the full set of filter outputs. Four clusters are sought, in keeping with the rotation of four PCs above. As with the PCA, the input to the k-means algorithm is the output of the five filters described in Table 3. A low-pass median filter with an influence area of 5 by 5 pixels is applied to the resulting cluster map to improve spatial coherence of the individual clusters.

### 3.4 Phenomena Identification

Once the individual images have been broken up into discrete regions with different spatial patterns of mesoscale near-surface wind speed variability, it is useful to determine which atmospheric phenomenon was responsible for each region. If the pattern recognition methods above are fully successful, each discrete region will be associated with a single atmospheric phenomenon, at least in the situation where no two phenomena overlap in space. Subjective analysis of several thousand SDWS images by the authors indicates that this is by far the most common situation, although overlap of island wakes and mountain waves do sometimes occur near mountainous coasts.

There are several approaches one can take to making this link between specific phenomena and individual regions of a SAR image. First, the analysis methods described above could be applied to the image set as a whole, following which each region (i.e. PC score pattern or cluster) would be manually matched with the corresponding phenomena by examination of the original images. This approach suffers from two major drawbacks, one computational and one meteorological. On the computational side, it requires that the filtered forms of all of the images be retained in memory at once. For the 1200 by 1200 pixel images used here, this is not practical. On the meteorological side, there are many possible atmospheric phenomena, so the analysis would need to be extended well beyond four clusters or PCs.

The second alternative for phenomena identification is to develop a categorical prediction system taking as input the statistics of a region in a particular image and returning as output the phenomenon responsible. Given enough training cases, a neural network [41] might provide the most robust results, but a simple Classification and Regression Tree (CART, [42]) is less likely to be over fit. This approach could be applied using either PC loadings or cluster centroids as input to the classification tree. Neither approach is tried here because the limited sample of 28 images provides too small a sample of each phenomenon. The third alternative, to manually identify the predominant phenomenon in each region can be used alone or as a check on this automated method. While manual phenomena identification is possible using only the raw SDWS images, it is easier following feature extraction by filtering, and easier still following pixel aggregation. As mentioned above, manual identification is tested at both the feature extraction and pixel aggregation stage. The results reported in the next section.

## 4. Results

Examination of the filtered SDWS images, PC score maps, and cluster maps was conducted for the 28 case study dataset designed to sample the more common mesoscale meteorological phenomena of the Gulf of Alaska (see Table 1). The overall results will be discussed via Table 5, which shows the fraction of cases of each phenomenon that were successfully highlighted by the various filters, the PCA, and the Cluster Analysis (the Sobel edge detection filter is not listed within Table 5 because of its relatively poor performance). These assessments were made manually by the authors who are seasoned SDWS analysts. At least half of the cases had multiple phenomena, as noted in Table 1, but for less than half of these multi-phenomena cases were any of the methods able to automatically distinguish between phenomena.

**Figure 3.**
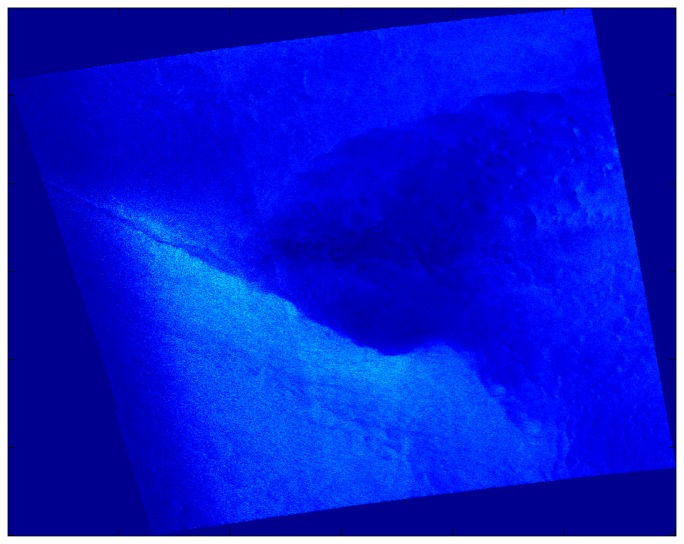
SDWS image for the case study, 0533 UTC on December 9, 2007. Brighter shades indicate higher wind speeds. The faint diagonal discontinuities parallel to the lateral borders of the image are artifacts resulting from satellite beam seams.

Performance characteristics of the individual methods are documented with the case study from 0533 UTC on December 9, 2007 and with particularly illustrative results from other cases. All images in this section are shown with north to the top of the page. Artifacts at the edges of the filtered images have not been removed. Figure 3 shows the SDWS image of the case study, with a front extending across the image at the northern edge of a band of large near-surface wind speed. Another, lesser front extends diagonally towards the northeast corner of the image. Between the two is an area of small near-surface wind speed, probably due to a seclusion of warm air, with the gust and lull patterns of convection cells (in this case, open cell convection [43]).

**Figure 4.**
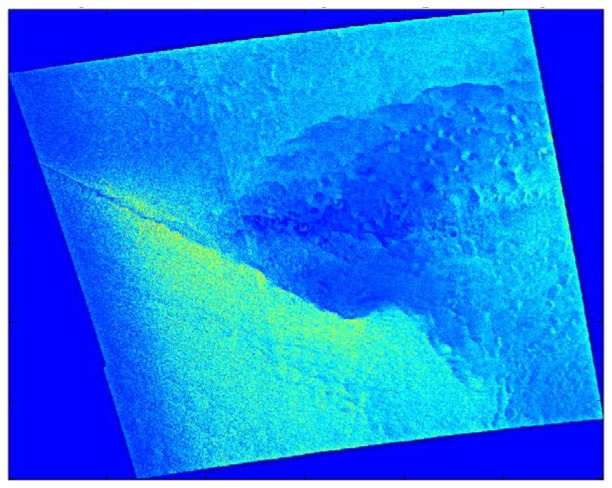
Low-pass Gaussian filtered SDWS image for the case study, 0533 UTC on December 9, 2007. Brighter shades indicate larger wind speeds with green being the largest.

**Figure 5.**
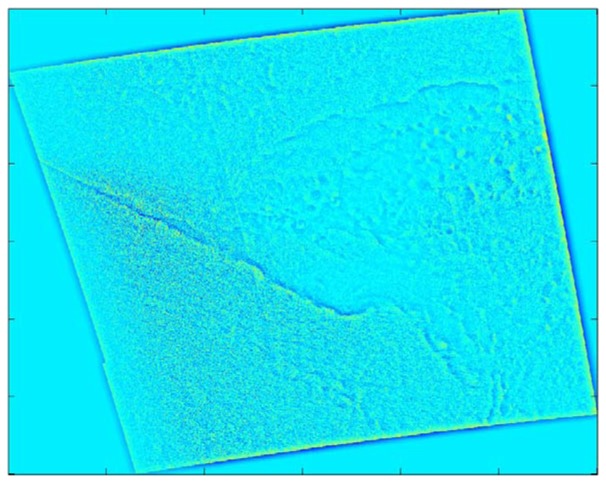
High-pass Gaussian filtered SDWS image for the case study, 0533 UTC on December 9, 2007. Brighter shades indicate larger wind speeds with green being the largest.

These features become much more apparent in the Gaussian low-pass filtered image (see Figure 4) because the microscale atmospheric and oceanographic variance (i.e. small-scale noise) have been reduced. The Gaussian high-pass filter also highlights these phenomena (see Figure 5), but does so by reducing the synoptic scale variance. The sharp gradients along the fronts and open cell squalls are depicted as narrow bands of green and adjacent dark blue within Figures 4 and 5.

Overall, for the 28-case sample, the Gaussian filters do the best job of highlighting the mesoscale phenomena, although the filter radius setting used here often splits the mesoscale near-surface wind speed variability between the high-pass and low-pass components. The low-pass images typically show the synoptic scale flow and a small-scale-noise-free view of the mesoscale phenomena. In contrast, the high-pass images show the mesoscale phenomena superimposed on a small-scale noise background. This suggests that the mesoscale phenomena would be captured more successfully with a band-pass filter having one cut-off between the synoptic and mesoscale (e.g. at around 50 km) and another between the mesoscale and microscale (e.g. at around 2 km). Even without this addition, the Gaussian filter was the most successful at highlighting gravity waves, convection cells, island wakes, and gap flow (see Table 4).

**Figure 6.**
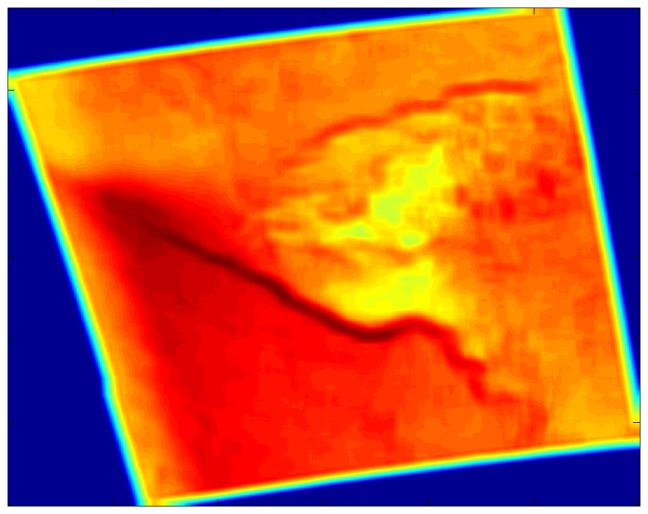
Local entropy filtered SDWS image for the case study, 0533 UTC on December 9, 2007. The two meandering darker red bands indicate the fronts. The seclusion appears as a green area in between.

**Figure 7.**
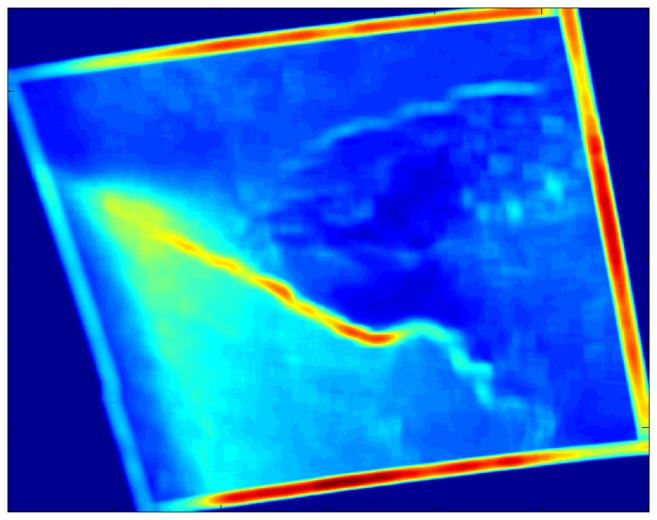
Local standard deviation filtered SDWS image for the case study, 0533 UTC on December 9, 2007. The two meandering bright blue to red bands indicate the fronts. The seclusion appears as dark blue speckled with the cyan rectangles corresponding to the mesoscale convective cells.

The local entropy and local standard deviation filters generally do a better job of highlighting the fronts than do the Gaussian filters, at least for the filter radius tested here (see Table 5). Figure 6 shows the local entropy filter output for the case study while Figure 7 shows the local standard deviation filter output. The fronts appear as narrow, sometimes meandering bands in these two filter outputs. Other features are less apparent, although the convection cells results in a characteristic pattern of rectangular splotches in the local standard deviation filter output. Interpretation of this convective signature is not as intuitive as that for the front, but it does appear in other open cell convective case as well.

**Figure 8.**
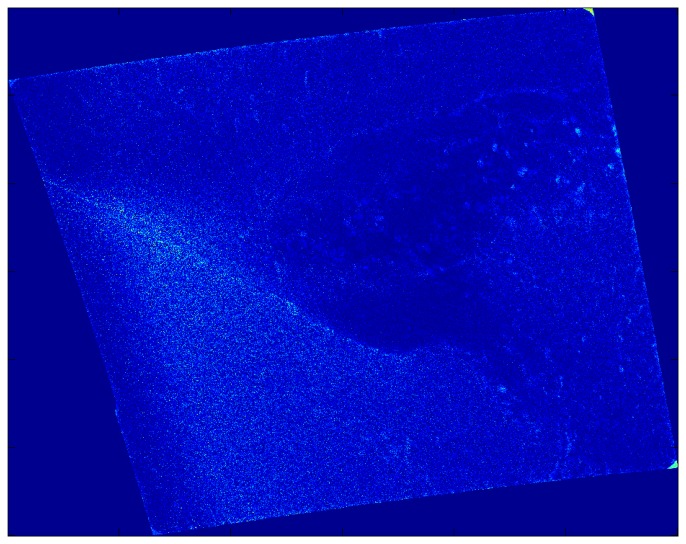
High-pass output of local median filter applied to the SDWS image for the case study, 0533 UTC on December 9, 2007. The two meandering bands of brighter blue speckles are the fronts.

The high-pass output of the local median filter typically captures only small-scale noise, although in the case study (Figure 8) it also highlights the fronts to some extent. As alluded to above, the Sobel edge detection filter (not shown) is even less successful, typically highlighting only the small scale noise in a SDWS image. The problem is that the edge detection filter highlights the very smallest (pixel-to-pixel) scales rather than separating these small-scales from those at which mesoscale near-surface wind speed variations occur. It may, however, help highlight image artifacts (e.g., beam seams) that could otherwise be mistaken for mesoscale near-surface wind speed variability.

**Figure 9.**
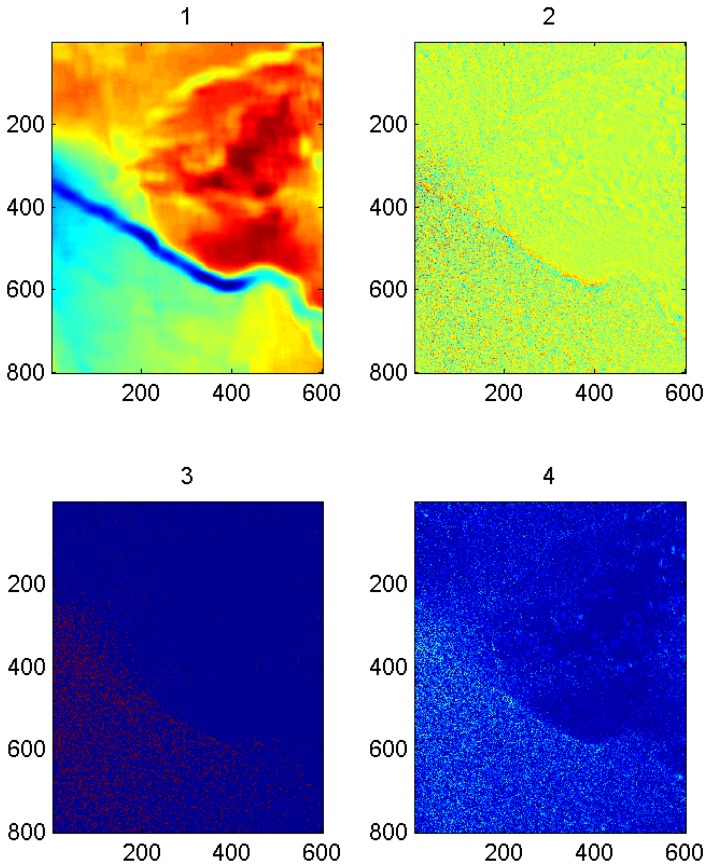
Each subimage is a Varimax rotated score map for the case study, 0533 UTC on December 9, 2007. The first shows the fronts as blue or yellow bands, the second shows the sharp edge of the strongest front as parallel blue and red lines. The third and fourth subimages show noise. The axes are labeled in pixel numbers.

For SDWS images with multiple mesoscale phenomena, the rotated PC score maps derived from the filter outputs generally provide better discrimination between phenomena than do the individual filters. The mesoscale phenomena usually appear on the first two rotated PCs while the third and fourth closely resemble the high-pass median and Sobel edge detection filter output. The first rotated PC frequently highlights the larger mesoscale phenomenon (e.g., the wakes of large islands) while the second rotated PC generally highlights the smaller mesoscale phenomena (e.g., gravity waves and the wakes of small islands). Thus, if only two mesoscale phenomena are present, one large and one small, rotated PCA serves to discriminate between the phenomena even when they are not separated in the raw filter outputs. If two phenomena occur on similar scales, however, they typically appear on the same PC score map. For the case study (see Figure 9), the fronts and the largest convection cells appear on the first PC score map while the second captures the smaller convection cells and the sharp edge of the most intense front. PC rotation is crucial to produce these scale separations, but both Promax and Varimax rotations produced similar results. Because the mesoscale near-surface wind patterns appear on only the first two rotated PCs and because the last two PCs closely resemble the median and edge filter outputs, a potentially useful enhancement of the PCA would be to exclude these two filter outputs from the PCA and to rotate only two of the resulting PCs.

**Figure 10.**
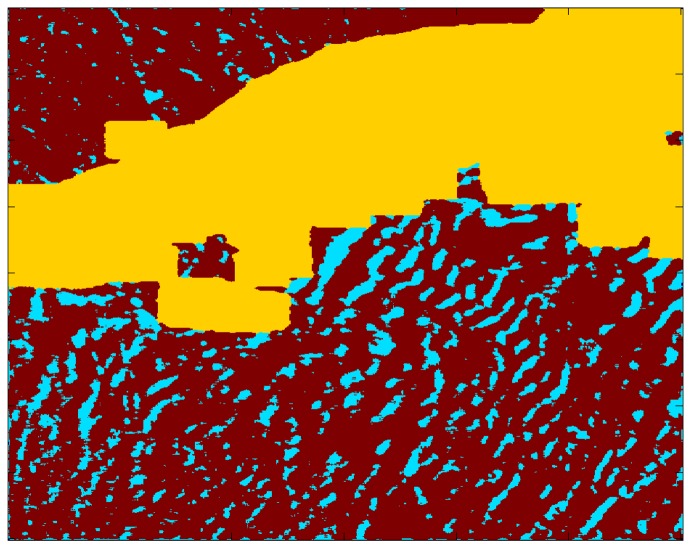
Despeckled cluster membership image for the case study, 0426 UTC on September 1, 2006. Each color represents points belonging to a common cluster. The fourth cluster was eliminated by the despeckling operation.

**Figure 11.**
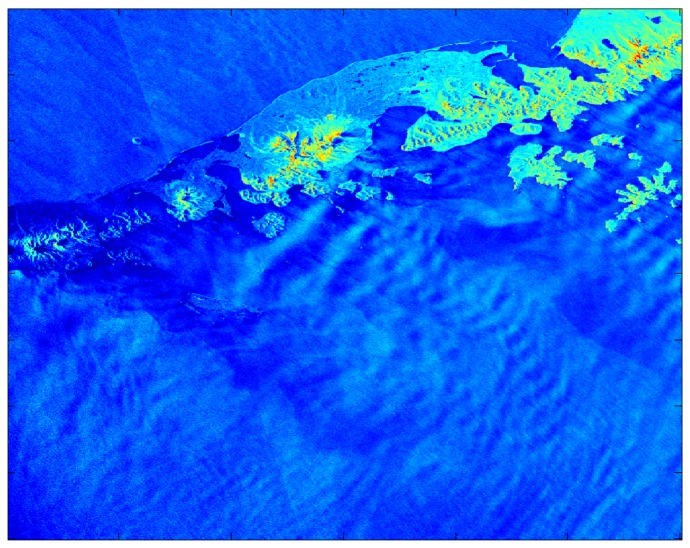
SDWS image for the case study, 0426 UTC on September 1, 2006. Brighter blue represents larger near-surface wind speed. Green represents land and yellow/orange/red represent mountains.

The cluster analysis, whether raw or despeckled, yields results that are similar to those of the PCA but generally not as successful. While the cluster analysis does not work well for the case study of 0533 UTC on December 9, 2007, it does for the case study of 0426 UTC on September 1, 2006. The despeckled cluster membership map for that SDWS image is shown in Figure 10 and the corresponding SDWS image in Figure 11. The yellow cluster highlights the masked-out land region (i.e. the Alaskan Peninsula and Aleutian Islands) while the red cluster captures the background near-surface wind speed and the cyan cluster the large near-surface wind speed areas corresponding to gravity wave troughs.

For the control (null) cases (No Significant Features within Table 1), performance of all filters is as one would expect: small-scale noise in the high-pass filter outputs and smooth synoptic scale near-surface wind speed patterns in the low-pass filter outputs. Thus, the filters are not synthesizing misleading patterns from the small-scale noise. As a result, the PCA yields equivalent results with the first score map showing the synoptic scale near-surface wind speed pattern and the other three showing small-scale noise. The cluster analysis also produces equivalent results with each cluster representing a particular near-surface wind speed band. Thus, the method defaults to showing the synoptic scale near-surface wind speed field if only that is present in the input SDWS image.

## 5. Summary and Conclusions

The Johns Hopkins University Applied Physics Laboratory, in conjunction with the National Ocean and Atmospheric Administration and the Alaska Satellite Facility, has produced an extensive archive of SAR-derived wind speed (SDWS) images (10 m above sea level). These images are available online at http://fermi.jhuapl.edu/sar/stormwatch/web_wind/. The variability of the near-surface wind speed observed within these images is often dramatic (e.g., change from breeze to gale/storm force over a distance of 1 to 10 km). Moreover, no other sensing system (remote or in situ) provides the ability to detect such variability over wide swaths of marine areas. Thus, SDWS images have the potential to be of great value to marine interests sensitive to such variability. The present research addresses this potential by taking the first steps toward automated detection of operationally-significant SDWS variability. Namely, the present research tests feature extraction (via digital filtering) and pixel aggregation (via PCA and cluster analysis) techniques focused on mesoscale near-surface wind speed variability. The meteorological features examined include gravity waves, convection cells, atmospheric fronts, island wakes, and gap flows.

Twenty eight SDWS case study images possessing varying degrees of near-surface wind speed variability were selected from the APL archive for use in exploring the performance of these techniques and developing recommendations for future research. After applying a land mask (when necessary) to each image, the SDWS images were subjected to Gaussian high- and low-pass, and high pass local entropy, local standard deviation, local median, and Sobel edge detection filters. The filtered images were then assessed manually. The Gaussian filters proved most beneficial in highlighting the mesoscale meteorological phenomena outlined above with the exception of atmospheric fronts, for which the local entropy and local standard deviation filters performed best. The local median and Sobel edge detection filters performed the worst for the task at hand, highlight small-scale near-surface wind speed variability rather than that of the mesoscale. Analysis of null cases shows that the filters do not produce misleading patterns from the small-scale noise.

The filtered output for each case was then used as input for the PCA and cluster analysis. Assessment was again manually. Promax- and Varimax-rotated PCA generally performed better than raw or despeckled cluster analysis for the cases studied. And, rotated PC 1 and PC 2 were the components that best highlighted the mesoscale phenomena. PC 1 tended to depict the larger mesoscale features (e.g., large island wakes) while PC 2 depicted the smaller scale features (gravity waves). PCs 3 and 4 tended to highlight microscale variability. For the null cases, PC 1 shows the synoptic scale near-surface wind speed pattern and the other three PCs show small-scale noise. Cluster analysis behaves similarly.

Recommendations for future studies include conducting PCA on only the output from the filters that performed best (Gaussian, local entropy, and local standard deviation). Moreover, because PC 1 and PC 2 routinely provided the best results, a two-component rotation (vice four) should be tested. For automated phenomenon detection, future studies should develop a categorical prediction system using the filter and PCA results as input. Given the large archive of SDWS images available at APL, a neural network may prove to be the best approach. These recommendations should undergo testing on this or a similarly large set of images.

## Figures and Tables

**Table 1.** Description of the case study image dataset used in this study. Column 1 includes the date/time information. Column 2 gives the general geographic location. Column 3 gives a description of any mesoscale features in the image that would be expected to be captured by the filtering. These features include meteorological phenomena as well as image artifacts that might impact the results. “Synoptic Scale Front” is included in this list because the sharp cross-front discontinuity is a major source of mesoscale near-surface wind speed variability.

**Image Date/Time**	**Location**	**Mesoscale Meteorological Phenomena**

2006, Sep. 01, 0426 UTC	Aleutians	Gravity Waves, Island Wakes
2006, Dec. 12, 1655 UTC	Aleutians	Convection Cells, Island Wakes
2007, Jan. 29, 0451 UTC	Aleutians	Island Wakes, Convection Cells
2007, Feb. 01, 0323 UTC	Gulf of Alaska	Image Artifact
2007, Feb. 05, 0446 UTC	Aleutians	Synoptic Scale Front, Gravity Waves, Island Wakes
2007, Feb. 25, 1527 UTC	Gulf of Alaska	Gap Flows, Image Artifact
2007, Feb. 26, 1819 UTC	Bering Sea	Synoptic Scale Front
2007, Apr. 29, 0426 UTC	Aleutians	Synoptic Scale Front, Gravity Waves, Island Wakes
2007, Sep. 20, 0425 UTC	Aleutians	Gravity Waves, Island Wakes
2007, Oct. 25, 0546 UTC	Bering Sea	Synoptic Scale Front and Cyclone
2007, Nov. 21, 0235 UTC	Gulf of Alaska	No Significant Features (Control Case)
2007, Dec. 07, 0310 UTC	Gulf of Alaska	No Significant Features (Control Case)
2007, Dec. 08, 0240 UTC	Gulf of Alaska	No Significant Features (Control Case)
2007, Dec. 09, 0533 UTC	Bering Sea	Synoptic Scale Front, Convection Cells
2007, Dec. 10, 0322 UTC	Gulf of Alaska	No Significant Features (Control Case)
2007, Dec. 11, 0252 UTC	Gulf of Alaska	No Significant Features (Control Case)
2007, Dec. 12, 0405 UTC	Aleutians	Synoptic Scale Front, Gravity Waves, Island Wakes
2007, Dec. 13, 0335 UTC	Gulf of Alaska	Stable Stratification Wind Speed Variability
2007, Dec. 18, 0250 UTC	Gulf of Alaska	Mesoscale Barrier Jet
2007, Dec. 21, 0301 UTC	Gulf of Alaska	Synoptic Scale Front, Gravity Waves
2007, Dec. 24, 0456 UTC	Bering Sea	Mesoscale Front
2007, Dec. 26, 0538 UTC	Bering Sea	Convection Cells
2007, Dec. 27, 0327 UTC	Gulf of Alaska	Synoptic Scale Front, Gravity Waves
2007, Dec. 27, 0507 UTC	Aleutians	Stable Stratification Wind Speed Variability, Island Wakes, Image Artifacts
2007, Dec. 28, 0258 UTC	Gulf of Alaska	Synoptic Scale Front, Gravity Waves, Image Artifacts
2007, Dec. 28, 1824 UTC	Bering Sea	Convection Cells, Image Artifacts
2007, Dec. 29, 0550 UTC	Bering Sea	Convection Cells, Stable Stratification Wind Speed Variability
2007, Dec. 30, 0340 UTC	Gulf of Alaska	Island Wakes, Gap Flows

**Table 2.** Feature extraction filters, the Matlab functions in which they are implemented, and the toolbox which contains them.

**Filter**	**Function**	**Toolbox**

Gaussian	filter2, fspecial	Core, Image Processing
Local Entropy	entropyfilt	Image Processing
Local Standard Deviation	stdfilt	Image Processing
Local Median	medfilt2	Image Processing
Sobel Edge Detection	edge	Image Processing

**Table 3.** Correlation matrix for the output of the edge detection and high-pass filters used in feature extraction. Each cell contains the mean and standard deviation over the set of 28 images.

N = 28 Images	**High-pass Gaussian**	**Local Entropy**	**Local Standard Deviation**	**Local Median**	**Sobel Edge Detection**
**High-pass Gaussian**	1±0	0.01±0.01	0.24±0.14	0.01±0.01	0.02±0.03
**Local Entropy**		1±0	0.40±0.15	0.90±0.15	0.18±0.08
**Local Standard Deviation**			1±0	0.39±0.13	0.08±0.05
**Local Median**				1±0	0.15±0.07
**Sobel Edge Detection**					1±0

**Table 4.** Feature aggregation algorithms, the Matlab functions in which they are implemented, and the toolbox which contains them.

**Algorithm**	**Function**	**Toolbox**

Principle Component Analysis	eig	Core
Component Rotation	rotatefactors	Statistics
K-means Cluster Analysis	kmeans	Statistics
Despeckling	medfilt2	Image Processing

**Table 5.** Fraction of the occurrences within the 28 case study images for which each mesoscale phenomena was enhanced relative to the background flow. Number of cases is in parentheses. Each row corresponds to one of the analysis procedures and each column to one of the more abundant atmospheric phenomena represented in the set of case study images.

	**Gravity Wave (8)**	**Convection Cells (6)**	**Synoptic Scale Front (9)**	**Island Wakes and Gap Flow (11)**

**Gaussian**	1.00	0.50	0.67	0.73
**Local Entropy**	0.00	0.17	1.00	0.44
**Local Standard Deviation**	0.00	0.17	1.00	0.44
**Local Median**	0.25	0.33	0.56	0.27
**PCA**	0.50	0.50	0.78	0.36
**Cluster**	0.50	0.17	0.44	0.36
